# Working Memory in the Service of Executive Control Functions

**DOI:** 10.3389/fnsys.2015.00166

**Published:** 2015-12-14

**Authors:** Farshad A. Mansouri, Marcello G. P. Rosa, Nafiseh Atapour

**Affiliations:** ^1^Department of Physiology, Monash UniversityMelbourne, VIC, Australia; ^2^ARC Centre of Excellence in Integrative Brain Function, Monash UniversityMelbourne, VIC, Australia; ^3^Neuroscience Program, Biomedicine Discovery Institute, Monash UniversityMelbourne, VIC, Australia

**Keywords:** executive control, prefrontal cortex, working memory, non-human primates, short-term memory

## Abstract

Working memory is a type of short-term memory which has a crucial cognitive function that supports ongoing and upcoming behaviors, allowing storage of information across delay periods. The content of this memory may typically include tangible information about features such as the shape, color or texture of an object, and its location and motion relative to the body, as well as phonological information. The neural correlate of working memory has been found in different brain areas that are involved in organizing perceptual or motor functions. In particular, neuronal activity in prefrontal areas encodes task-related information corresponding to working memory across delay periods, and lesions in the prefrontal cortex severely affect the ability to retain this type of memory. Recent studies have further expanded the scope and possible role of working memory by showing that information of a more abstract nature (including a behavior-guiding rule, or the occurrence of a conflict in information processing) can also be maintained in short-term memory, and used for adjusting the allocation of executive control in dynamic environments. It has also been shown that neuronal activity in the prefrontal cortex encodes and maintains information about such abstract entities. These findings suggest that the prefrontal cortex plays crucial roles in the organization of goal-directed behavior by supporting many different mnemonic processes, which maintain a wide range of information required for the executive control of ongoing and upcoming behaviors.

## Short-Term Storage of Information Required to Guide Ongoing or Upcoming Behavior

The concept of working memory describes a process of short-term storage of information to support ongoing or upcoming actions, and is considered a crucial component of the executive control of goal-directed behavior (Baddeley, 1986; Fuster, 1995; Goldman-Rakic, 1995a,b). One view, emerging mostly from human studies, considers working memory as an essential intermediate stage (or buffering system) for retrieved memories, thus enabling further manipulation and integration of information involved in perceptual and mental functions (Baddeley, 1986, 2012). A related perspective, mostly focused on the neural substrate of working memories, assumes that retention of task-relevant information is essential for complex behaviors which evolve in time, in order to maintain the perception and actions in a coherent and goal-directed framework. Therefore, working memory processes appear crucial for the temporal organization of behavior (Fuster, 1997; Fuster et al., 2000), including linking processes across delays (Goldman-Rakic, 1995a,b). Related models propose that other short-term memory functions provide an intermediate stage for the buffering and exchange of information between working memory and long-term memory repositories (Ericsson and Kintsch, 1995).

Various techniques, including non-invasive imaging and cellular and molecular studies in animal models, have enriched our knowledge about the working memory process. Here, we briefly review some of the studies that have been conducted in non-human primates to examine the neural substrates and mechanisms of working memory, with emphasis on recent work that demonstrates working memory for abstract features such as rules and strategies.

## Working Memory in Non-Human Primates

Single-cell recordings afford high temporal and spatial resolution for the study of information conveyed by neuronal activity. This type of research, using behaving monkeys, has provided ample evidence for the involvement of different cortical and sub-cortical areas in the short-term storage of information in delayed response tasks. In such studies the cognitive tasks typically include an encoding period, during which a to-be-remembered “cue” or “sample” is presented, followed by a delay period, during which information about the cue has to be maintained for successful resolution of an upcoming problem. At the end of the delay period the memory of the cue is tested by requiring an operant behavior to select a choice. Examples of cognitive tasks with such paradigms include the delayed matching to sample task, in which a choice object that matches the sample needs to be selected, and the delayed alternation task, in which an alternative action, different from a previous response, has to be selected (Fuster, 1995; Goldman-Rakic, 1995a,b, 1996). Various tasks have examined the process of working memory in different modalities (such as visual, auditory or tactile) by changing the features and modality of the to-be-remembered cue. Neural correlates of working memory have been found in many different brain areas, including those typically regarded as being involved in perceptual and motor functions.

## Working Memory of Concrete Entities

In a classical study, Fuster and Alexander (1971) trained monkeys to perform a delayed response task in which the monkeys had to remember a visual cue across a delay period. The authors found that a significant number of cells in prefrontal cortex and in the mediodorsal nucleus of thalamus displayed a persistent increase in activity during the delay period. This led them to conclude that this persistent activity might represent the mnemonic processes that enable short-term storage of information across the delay period. Kubota and Niki (1971) also reported persistent activity during the delay period in the context of a delayed alternation task. These pioneering studies supported the emerging idea that working memory is based on maintained representation of events and stimuli, even after their cessation, in the prefrontal neurocircuitry. Fuster (1990, 1995, 1997) subsequently suggested that such representations enable temporal linking of recent salient experiences to the upcoming action. These studies were followed by others which characterized the relationship between the delay-period activities, the preceding (to-be-remembered) stimulus features, and the intended (upcoming) action, as well as the persistence of this activity and its resistance to distraction and interruption.

In another study, Funahashi et al. (1989) examined the delay period activity in a more controled condition, in which eye position was closely monitored and the monkeys were required to maintain information of a location in space, to guide an upcoming saccadic eye movement. Eye fixation during the delay period was crucial to rule out the possible confounds arising from different eye positions during the delay period. Their findings revealed the presence of “memory fields” within the prefrontal cortex, suggesting that separate memory-processing modules covered the visual scene in terms of temporary storage of memory. They also showed that the delay period activity was attenuated in error trials (in which the eye saccade was made erroneously in a manner that was unrelated to the previously given information), suggesting that the delay period activity was linked to correct behavioral performance. This finding was first to link the persistent delay period activity to the overall behavior of the monkeys. In follow-up studies, the same group (Funahashi et al., 1993a,b) provided evidence to support the idea that a memory map in prefrontal cortex underlies spatial working memory. However, related studies indicated that sustained activity in the delay period was not a unique property of prefrontal neurons. Cellular activity in other cortical areas, particularly the posterior parietal cortex, also conveys information during delay periods (Gnadt and Andersen, 1988; Chafee and Goldman-Rakic, 1998). These findings raised important questions regarding the significance of delay period activity in guiding overall behavior, its relation to required mnemonic process and other impending processes or actions, and the possible differential contributions of individual brain areas to the working memory process.

In the following years different research groups found sustained neuronal activity in delayed response tasks in various compartments of the prefrontal cortex as well as in the sensory and motor areas (di Pellegrino and Wise, 1991; Miller et al., 1993; Ferrera et al., 1994; Motter, 1994; Bodner et al., 1996; Constantinidis and Steinmetz, 1996; Miller et al., 1996; Rao et al., 1997; Asaad et al., 1998; Chelazzi et al., 1998; Rainer et al., 1998a,b; Romo et al., 1999; Fuster et al., 2000; Zaksas et al., 2001; Pardo-Vazquez et al., 2008, 2009; Rawley and Constantinidis, 2009; Sigala, 2009). These studies showed that, depending on the task demand, information about different stimulus features, from different modalities, could be maintained in working memory and represented in neuronal activity within the prefrontal cortex and sensory areas.

In a landmark study, Rao et al. (1997) trained monkeys to perform a delayed response task in which they had to make a saccade to the remembered location of an object. In each trial, the object was presented briefly at the center of the screen, and then replaced by a fixation point. During the ensuing delay period the monkeys had to retain information about the identity of this particular object (sample) in their short-term memory. Two different objects were then shown, one of which matched the previously presented sample. This was followed by another delay period (in which the monkeys had to hold information regarding the “sample location”) before the appearance of four saccade targets on the screen; only then did the animals make saccades to the remembered location of the object. Therefore, in the same trial the monkeys had to retain the memory of an object and its location in two separate delay periods, respectively. This study showed that the same population of prefrontal neurons can convey information about objects and their locations, across two delay periods, depending on the task demands. Such neurons were distributed in different parts of the lateral prefrontal cortex, indicating that representations of working memory of objects and their locations are not regionally segregated.

These findings have changed the classic view of the prefrontal cortex as the powerhouse of working memory processes. Recent models suggest that short-term storage of discrete information can be achieved in the same areas that initially process the sensory information and enable perception (Pasternak and Greenlee, 2005; Zaksas and Pasternak, 2006; Lui and Pasternak, 2011; D’Esposito and Postle, 2015). An important question arising from these studies is the specific contribution of prefrontal cortex to these mnemonic processes. Different models have emerged from imaging and animal model studies to suggest that the storage of information in short-term memory can be accomplished by sensory areas; however, persistent representations in prefrontal cortex might act as a medium for additional processes on the maintained representation of stimuli, as well as the application of these to guide goal-directed behavior (Pasternak and Greenlee, 2005; D’Esposito and Postle, 2015). This view is supported by numerous studies showing that cellular activity in the prefrontal cortex during cue-presentation and/or delay period activity can convey information about the upcoming reward (Watanabe, 1986, 1996; Watanabe et al., 2002; Leon and Shadlen, 1999; Tremblay and Schultz, 2000; Kobayashi et al., 2002; Wallis and Miller, 2003), the upcoming actions (Quintana and Fuster, 1992; Asaad et al., 1998; Ferrera et al., 1999; Hoshi et al., 2000) and the task context (Sakagami and Niki, 1994; Hoshi et al., 1998; White and Wise, 1999; Wallis et al., 2001; Barraclough et al., 2004; Genovesio et al., 2005; Johnston and Everling, 2006; Mansouri et al., 2006). The findings of neuroimaging and neuropsychological studies in humans also support this emerging view regarding the contribution of the prefrontal cortex to primate cognition (Sakai and Passingham, 2003, 2006; Müller and Knight, 2006; Sreenivasan et al., 2014).

In summary, our views about the function of the prefrontal cortex as a center for working memory of task-relevant information has evolved to a more comprehensive model, which considers the prefrontal cortex as the site of dynamic and highly plastic integrative machinery for the executive control of behavior. Such integrative functions are supported by reciprocal connections between the prefrontal cortex, sensory association areas, premotor areas, and areas involved in the organization of emotions and motivations (Barbas, 2000; Burman et al., 2011, 2015; Petrides et al., 2012; Reser et al., 2013). These connections might enable prefrontal areas to select sustained neural representations in sensory areas, and link them to other task-relevant information such as reward and actions and/or retrieved memories, in order to construct an active representation of the task set required to achieve a particular goal (Miller, 1999; Miller and Cohen, 2001; Courtney, 2004; Deco and Rolls, 2005; Pasternak and Greenlee, 2005; Ranganath, 2006; Watanabe and Sakagami, 2007; Rushworth et al., 2011; Funahashi and Andreau, 2013; D’Esposito and Postle, 2015).

## Working Memory of Abstract Entities Within and Across Trials

Other studies have shown that the information contained in working memory can be of a more abstract nature. Nieder et al. (2002) and Nieder (2005) trained monkeys to perform a delayed matching to “number of items” task, in which the monkeys first observed a sample comprising several items; after the delay period they then had to decide whether the display had the same number of items. The exact physical appearance of the displays was changed, and the monkeys therefore had to maintain information about “numerosity” during the delay period. The authors found that prefrontal cell activity encoded and maintained such information, suggesting that the abstract concept of number can be held in working memory via prefrontal neurons.

In another series of studies Mansouri and Tanaka (2002) and Mansouri et al. (2006, 2007, 2014) trained monkeys to perform a computerized analog of Wisconsin Card Sorting Test (WCST; Figure 1A). In the WCST, successful adaptation to the unannounced rule changes requires maintenance of the information about the relevant rule within and across trials. The monkeys had to match a sample to one of three test items based on either color or shape. A liquid reward and a discrete visual signal (error signal) were given as feedback to correct and incorrect target selections, respectively. The relevant rule and its frequent changes were not cued, meaning that the monkeys could find it only by interpreting the feedback. These studies showed that monkeys can successfully perform the WCST analog, indicating that they could infer and memorize the relevant rule. A significant number (about 30%) of dorsolateral prefrontal neurons near the principal sulcus represented the rules within and across trials, independent of the other aspects of the task (Figure 1C). The magnitude of the rule-dependent activity modulation correlated with the number of errors that the monkeys made after each rule change, in the course of reestablishing high performance. This indicated a link between representation of the working memory of the rules and the efficiency of the monkeys’ overall behavior in adapting to frequent rule changes. However, information regarding the rule was retained in prefrontal cell activity during error trials, when the monkeys used the irrelevant rule to guide their behavior. This suggested that even during error trials information about the relevant rule was maintained in the prefrontal neurocircuitry, but for some other reasons such as a lapse of attention, or inaccessibility of the content of working memory for the decision process, the monkeys did not follow the relevant rule (Mansouri et al., 2006). Follow-up studies showed that lesions within the dorsolateral prefrontal cortex, orbitofrontal cortex or anterior cingulate cortex impaired performance of the WCST analog (Buckley et al., 2009; Kuwabara et al., 2014).

**Figure 1:**
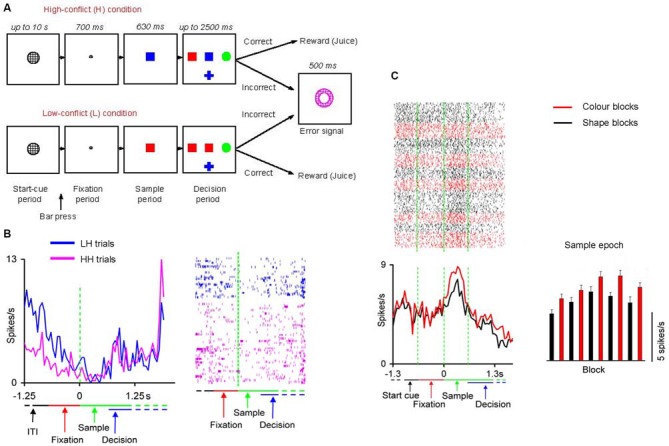
**Neuronal activity in prefrontal cortex representing abstract entities. (A)** Cognitive task paradigm. In each trial, a start cue (a gray circle) appeared when an inter-trial interval (ITI) was over. The monkey had to push a bar after the onset of the start cue. This action changed the start cue to a fixation point, after which a sample stimulus replaced the fixation point. If the monkey maintained eye fixation and bar press three test items appeared (to the left, right and below the sample). The monkeys had to touch the test item that matched the sample in color or shape. The relevant rule for matching (matching by shape or matching by color) was consistent within a block of trials. The relevant rule was not cued and changed without any notice to the monkey when a criterion of 85% correct performance was achieved. **(B)** Dorsolateral prefrontal cortex cell activity represented conflict level experienced in the previous trial. The rastergram indicates activities in individual correct trials. Each row corresponds to a trial and each dot represents an action potential. Activities in high-conflict trials after low-conflict trials (LH, blue) and those in high-conflict trials after high-conflict trials (HH, pink) are shown. The mean activities are aligned at sample onset. **(C)** Activity difference between color and shape blocks in a dorsolateral prefrontal cortex cell represented the matching rules. The line graphs on the bottom left show the averaged firing rates in color and shape blocks, aligned at the sample onset. The bar graph on the bottom right represents the mean firing rate during the Sample epoch in consecutive blocks. The red and black dots, lines, and bars indicate color and shape blocks, respectively. The bin size is 50 ms. **(A,B)** are adapted from Mansouri et al., 2007 (Ref. 49). **(C)** is adapted from Mansouri et al., 2006 (Ref. 48).

Additional studies examined the susceptibility of the working memory of the relevant rule to changes in task demand and interruptions. After the monkeys reached a high performance level with a particular rule, the inter-trial interval (ITI) was lengthened to increase the period during which the memory had to be held across trials. The monkeys’ ability to remember the relevant rule was then tested in the following trial. The working memory of the rule was very vulnerable to changes in the holding period, as the performance of control monkeys (without a brain lesion) significantly decreased after the long ITI, although it still remained above the level of chance (Buckley et al., 2009). Monkeys with lesions of the dorsolateral prefrontal cortex were the most susceptible to this manipulation and their performance dropped to the chance level, whereas animals with orbitofrontal or anterior cingulate lesions could still perform the WCST above the chance level (Buckley et al., 2009). Mansouri et al. (2014, 2015) also examined the vulnerability of the working memory process to interruptions. Working memory of the rule was very vulnerable to distractions as introducing salient events such as free reward or performing a simple additional task during the ITI completely disrupted working memory and the performance dropped to the chance level in control monkeys.

These findings indicate that working memory processes maintain abstract information, and are not limited to a single trial, bridging the ITI to maintain the information that is necessary to guide behavior in the following trials. Other studies have also shown that information of task/rule might be maintained in prefrontal cell activity within and across trials (Rainer et al., 1998b; Asaad et al., 2000; Wallis et al., 2001).

## Mnemonic Processes in Context-Dependent Executive Control Adjustment

Conflict in information processing and the occurrence of errors evoke trial-by-trial modulations in behavior. It has been proposed that adaptive tuning of executive control, mediated by the dorsolateral prefrontal cortex, underlies these modulations (Botvinick et al., 2001; Carter and van Veen, 2007; Egner, 2007; Mansouri et al., 2009; Schroder and Infantolino, 2013; Wessel et al., 2014). The behavioral modulations induced by conflict and error are seen in the trial in which these first become manifest, and also in subsequent trials. It has been suggested that a mnemonic process is necessary to modulate behavior according to conflict experienced in an earlier trial, so that the required information is maintained (Mansouri et al., 2007, 2009). When the conflict-inducing task context ends, this mnemonic process should hold information about conflict during ITIs, to enable modulation of behavior in upcoming trials (Mansouri et al., 2009).

To examine the neural substrate and underlying neural mechanisms of conflict-induced behavioral adjustment, Mansouri et al. (2007, 2009) trained monkeys to perform a version of the WCST in which the level of conflict changed trial-by-trial. The monkeys’ behavior was modulated by conflict in the current and following trials, and neuronal activity in dorsolateral prefrontal and orbitofrontal cortices encoded the existing conflict level. Another group of cells in the dorsolateral prefrontal cortex modulated their activity during the ITI depending on the conflict level in the previous trial, but such neurons were not observed in the orbitofrontal cortex (Mansouri et al., 2007, 2009, 2014; Figure 1B). This activity modulation may represent a mnemonic process that maintains information of conflict across trials. Modulation of behavior by an error in an earlier trial might also require such a mnemonic process during the ITI, and previous studies have shown that the activity of dorsolateral prefrontal cortex (Mansouri et al., 2006) and orbitofrontal cortex cells (Simmons and Richmond, 2008) maintains information of errors during the ITI.

These studies suggest that different compartments of the prefrontal cortex make dissociable contributions to mnemonic processes in the performance of the WCST. Compared to the consequence of lesions in other prefrontal and medial frontal regions, lesions in the principal sulcus led to the most significant impairment of the mnemonic processes (Mansouri et al., 2007, 2014, 2015; Buckley et al., 2009), These findings suggest that the dorsolateral prefrontal cortex might be more involved in working memory processes in the WCST. Nieder et al. (2002) and Eiselt and Nieder (2015), showed that neuronal activity in dorsolateral prefrontal and parietal areas, but not in premotor or cingulate motor areas, encodes numerosity information during sample and working memory periods, suggesting that working memory of numerosity is supported by the dorsolateral prefrontal cortex and parietal cortex.

## A Broader Perspective of Working Memory

Historically (Funahashi et al., 1989; Fuster, 1995; Goldman-Rakic, 1995b; Miller and Cohen, 2001; Constantinidis and Procyk, 2004; Deco and Rolls, 2005; Pasternak and Greenlee, 2005; Ranganath, 2006; Cowan, 2008; Baddeley, 2012), a number of features have been described for working memory: (i) it has a short duration, and fades as the delay period gets longer; (ii) it is goal-oriented and its content is used to guide upcoming behavior; (iii) it is limited to a trial, being updated in each subsequent trial; (iv) it is highly vulnerable to distraction; (v) its content is a discrete feature of an object or event such as a particular color, shape or position in space; and (vi) subjects intentionally store information in working memory to solve a problem and are therefore aware of its content.

Recent studies suggest that prefrontal cortex also supports a kind of memory that maintains information about task context, in order to enable context-dependent executive control adjustment in subsequent trials. This mnemonic process shares some aspects of the concept of working memory defined in delayed response tasks in that: (i) it maintains task-relevant information for a short period; (ii) its content, which could be an abstract variable such as conflict, is updated trial-by-trial; and (iii) it is crucial for optimizing performance in a goal-directed task. However, this memory also differs from working memory in that maintaining the information is not intended and the subjects can still perform the task, although not optimally, without such information.

## Conclusion

Working memory is essential for the organization of goal-directed behavior, as it maintains task-relevant information. Sustained delay period activities in prefrontal cortex have been traditionally considered as neural mechanisms for encoding the working memory. However, four decades of studies on working memory indicate that this is not a unique property of the prefrontal cortex neurocircuitry, and that distributed networks including sensory systems and sub-cortical areas are also involved in the short-term storage of information. In addition, converging evidence from various experimental approaches indicates that the prefrontal cortex might selectively combine sustained representations of task-relevant events with information such as task goal, behavioral rules, conflict and actions to construct a representation of the goals and strategies required to achieve these goals.

Recent studies suggest that various kinds of short-term memories maintain task-relevant information such as errors and conflict to enable adaptive adjustments in the executive control of behavior. These are mnemonic processes in the service of executive control to optimize behavior, based on recent experiences. Prefrontal cortex cells represent these mnemonic processes, and lesions within the prefrontal cortex impair the adaptive behaviors that are dependent on these processes. The concept of working memory could be broadened to include these short-term memories that are not directly necessary to perform the task, but are used to optimize performance. During the performance of goal-directed behaviors, parallel and diverse mnemonic processes, distributed in multiple networks, might actively maintain task-relevant information to enable a rich representation of goals, actions, rules and strategies at different levels of abstraction. The prefrontal cortex could therefore play a unifying role in linking these diverse but relevant processes to optimize the use of the cognitive resources that are necessary to control the goal-directed behavior.

## Funding

This study was supported by strategic grant scheme program, School of Biomedical Sciences at Monash University and ARC Centre of Excellence for Integrative Brain Function at Monash University.

## Conflict of Interest Statement

The authors declare that the research was conducted in the absence of any commercial or financial relationships that could be construed as a potential conflict of interest.
